# Good cognitive performances in a child with Prader-Willi syndrome

**DOI:** 10.1186/1824-7288-39-74

**Published:** 2013-11-15

**Authors:** Rosa Nugnes, Eugenio Zito, Enza Mozzillo, Maria Erminia Camarca, Maria Pia Riccio, Gaetano Terrone, Daniela Melis, Carmela Bravaccio, Adriana Franzese

**Affiliations:** 1 Department of Translational Medical Sciences, Federico II University of Naples, Italy, Via Pansini, 5-80131 Naples, Italy; 2 School of Movement Sciences (DiSiST), Parthenope University of Naples, Naples, Italy; 3 Department of Physical and Mental Health and Preventive Medicine, Second University of Naples, Naples, Italy

**Keywords:** Prader-Willi syndrome, Uniparental disomy (UPD), Cognitive profile, Behavior, Intellectual quotient (IQ)

## Abstract

We report the case of a child affected by Prader-Willi syndrome (PWS) with good cognitive performances and without relevant behavioral abnormalities.

The diagnosis of PWS, suspected on the basis of clinical features and past history, was confirmed by DNA methylation analysis. Additional genetic testing revealed a maternal uniparental disomy. Intellectual profile was analyzed by WISC-III and Raven’s Progressive Matrices CPM, while the behavior was evaluated by K-SADS-PL and Child Behavior Checklist/4-18 to the parents.

WISC-III test showed a Total Intelligence Quotient (T-IQ = 79) at the border level for age. The Verbal Intelligence Quotient (V-IQ) showed a lower score than the Performance Intelligence Quotient (P-IQ) (78 and 85, respectively). Raven’s Matrices CPM showed an intelligence level at 75-90° percentile for age. Concerning behavioral evaluation, a difficulty in impulse control was observed, with persistent but controllable search for food, without a clear psychopathological meaning. Also according to K-SADS-PL no areas of psychopathological dimensions were detected. In conclusion, in presence of consisting clinical features of PWS and high diagnostic suspicion, the diagnosis of PWS should be considered even in presence of a borderline IQ and in absence of psychopathological abnormalities.

## Background

Prader-Willi syndrome (PWS) is a complex genetic disorder, characterized by endocrine, neurologic, cognitive and behavioral abnormalities. Prevalence is about 1/15.000-30.000 [1], without differences among genders and ethnic groups. Infants with PWS show marked hypotonia, early feeding problems, difficulty in weight gain, dysmorphic features and genital abnormalities. During childhood, patients develop hyperphagia, morbid obesity, short stature, hypogonadism, global development delay, cognitive and behavioral disorders.

PWS is caused by the lack of expression of paternally derived alleles of imprinted genes located in the 15q11-q13 chromosome region; these genes are physiologically silenced on the maternally inherited chromosome. Genetic defects responsible for PWS are ‘de novo’ deletion in the paternal chromosome in 70-75% of patients, maternal uniparental disomy (UPD) in 20-25% of patients, and imprinting center defects in the remaining 1-5% of cases. Diagnosis can be suspected from clinical features and confirmed by genetic testing. DNA methylation analysis is the initial investigation; if positive, additional testing (FISH, karyotype and DNA polymorphism analysis) should be performed on the proband and parents to distinguish the genetic subtype and perform appropriate genetic counseling [2,3].

We report a case of a child affected by Prader-Willi syndrome (PWS) with cognitive performances slightly different from the classical intellectual functioning observed in these patients, and without relevant behavioral abnormalities.

## Case presentation

We describe the case of A.M., 8 years old child, who lived in Venezuela for the first 7 years of life, before moving to Italy. Clinical history is characterized by hypotonia, poor suck and cryptorchidism at birth, and successively by motor delay. He practiced physiotherapy with improvement of motor activity. Cryptorchidism was corrected by orchidopexy. Since the age of 2 years, he started to increase his daily caloric intake and became progressively obese. A.M. was referred to our outpatient clinic at the age of 7 years for severe obesity with a Body Mass Index (BMI) of 30.6 Kg/m^2^ (weight: 42 kg; height: 117 cm). He also presented low forehead, almond-shaped eyes, thin upper lip, high-arched palate, small hands, tapering fingers and hepatomegaly. Blood exams were normal. An abdomen ultrasound scan revealed the presence of fatty liver disease. EEG and ocular fundus were normal. The clinical features and his history led to suspect PWS, confirmed by DNA methylation analysis. Additional genetic testing revealed a maternal uniparental disomy (UPD). Physiatrist evaluation highlighted the presence of a normal motor activity, not needing physiotherapy. Intellectual profile was analyzed by WISC-III and Raven’s Progressive Matrices CPM, while the behavior was evaluated by K-SADS-PL and Child Behavior Checklist/4-18 to the parents (Table 1). There wasn’t any cognitive impairment, speech was fluent, articulate, it was also evident the presence of bilingualism (Italian and Spanish languages), good interpersonal and appropriate adaptive skills to the context of assessing. A good contact with reality and a good space-time orientation emerged. Obesity management was assessed by the institution of a well-balanced low-calories diet and by an appropriate psychological and behavioral counseling of the patient and family. The patient showed a good dietetic compliance and he managed to keep under control hunger. Indeed, after a twelve months follow-up, we observed a significant weight loss, with a decrease of BMI from 30.6 to 22.3 Kg/m^2^. Finally, despite the change of socio-cultural environment, familiar and social relationships were satisfying, and the school placement was adequate (he was learning a third language!).

**Table 1:** Assessment of intellectual profile and behavior in our patient

**Explored areas**	**Tests**	**Results**
Intellectual profile	WISC-III	Verbal IQ 78 (CI 73–87)
		Performance IQ 85 (CI 78–94)
		Total IQ 79 (CI 74–86)
	Raven’s Progressive Matrices (CPM)	Intelligence 75°- 90° percentile (higher than average)
Behavior	K-SADS-PL	Normal
	Child Behavior Checklist CBCL - 4/18	Normal

Summarizes the results of the PWS patient to the tests WISC-III and Raven’s Progressive Matrices (CPM) for the assessment of intellectual profile and to K-SADS-PL and Child Behavior Checklist CBCL - 4/18 for the assessment of behavior.

PWS patients usually present learning disabilities, poor school performance, temper tantrums, and psychotic disorders, increasing with age and BMI [1]. Indeed, several studies on large samples of PWS patients report the presence of a global intellectual abilities impairment of these children. However, according to recent studies, about 10-25% of PWS patients show normal or borderline levels of intellectual functioning [4,5].

WISC-III test administered to our patient showed a Total Intelligence Quotient (T-IQ = 79) at the border level for age. The Verbal Intelligence Quotient (V-IQ) showed a lower score than the Performance Intelligence Quotient (P-IQ) (respectively 78 and 85), probably depending on the adaptation to the new socio-cultural context. According to this hypothesis, the sub-tests “Vocabulary” and “Comprehension” of the verbal score, strongly related to the learning of a new language, showed slightly low scores (Table 2). The performance profile, however, showed mild difficulties in sustained attention and visual-motor dexterity by subtests “Coding” and “Symbol search” (Table 2), but globally the level of performance remained within the normal range. In order to remove the confounding socio-cultural factors, intellectual assessment was integrated by Raven’s Matrices CPM, which showed an intelligence level at 75-90° percentile for age.

**Table 2:** Weighted scores at WISC-III

**Subtests**	**Verbal**	**Performance**
Picture completion		10
Information	7	
Coding		4
Similarities	9	
Picture arrangements		9
Arithmetic	7	
Block design		8
Vocabulary	6	
Object assembly		8
Comprehension	5	
(Symbol search)		5
(Digit span)	5	
(Mazes)		7

Contains the weighted scores at the 13 subtests of WISC-III, administered to the PWS patient.

Concerning behavioral evaluation, a difficulty in impulse control was observed, with persistent but controllable search for food, without a clear psychopathological meaning. According to K-SADS-PL and CBCL to parents, no areas of psychopathological dimensions were detected. However, it is to be considered the risk of a bias in the parents information because the hope of a normal development of their child could lead them to minimize the symptoms. In addition, it is important to remark that our patient is a child and the risk of behavioral and psychopathological abnormalities mainly increases in adolescents and young adults affected by PWS [6].

Since many studies have claimed the presence of a genotype-phenotype correlation in PWS, we hypothesized that the border intellectual and behavioral profile of our patient could be related to the genetics. In literature, many studies underline the presence of differences in cognitive and behavioral aspects between patients with different genetic subtypes. Concerning the cognitive aspects, most studies have not found significant differences between “deletion” and “UPD” genotypes in T-IQ [5,7], while differences have been noted in V-IQ and P-IQ. Indeed, the “deletion genotype” is associated with better P-IQ scores, while the “UPD genotype” with better V-IQ scores [4,7-9]. However, our patient showed a V-IQ lower than P-IQ despite carrying a “UPD genotype”. We believe that this result was related to the acquisition of a new language after migration from Venezuela. Concerning the behavioral aspects, the “deletion genotype” has been associated more frequently with self-injurious skin picking and obsessive-compulsive and ritualistic behaviors [8,10], while the “UPD genotype” has been associated with a higher risk of autistic spectrum and psychotic disorders [11,12]. Moreover, patients carrying UPD seem to have less maladaptive behaviors compared to patients with deletions. Our patient did not show relevant behavioral abnormalities so far, but this cannot be interpreted as a probe that he could evolve to a complete autonomous normality in adolescence and young age. Moreover, given the reported association of “UPD genotype” with autistic spectrum and psychotic disorders, a special attention will be addressed to these aspects in the follow-up of our patient.

## Conclusions

PWS is a complex genetic disorder usually characterized by a global intellectual abilities impairment, learning disabilities, poor school performance. Although most of PWS patients show mild to moderate retardation, a lower percentage of about 10–25% displays a normal or borderline functioning, as in the case described. In addition, PWS includes behavioral and psychopathological abnormalities that mainly increase in adolescence and young age. In light of these observations, we conclude that, in patients with suggestive clinical features and history, diagnosis of PWS should be considered even in presence of a normal or borderline IQ and in absence of a clear psychopathological profile, particularly when the patient under examination is still a child. On contrary, in order to avoid unnecessary expensive and stressful diagnostic investigations, it is important to carefully evaluate all the clinical features of the patient, to integrate clinical evaluations of all the physicians of the multidisciplinary team assessing the patient, and to request molecular investigation only when there are consisting clinical data of PWS diagnostic suspicion.

## Consent

Written informed consent was obtained from the patient’s parents for publication of this case report and any accompanying images. A copy of the written consent is available for review by the Editor-in-Chief of this journal.

## Abbreviations

PWS: Prader-Willi syndrome; UPD: Uniparental disomy; BMI: Body Mass Index; T-IQ: Total Intelligence Quotient; V-IQ: Verbal Intelligence Quotient; P-IQ: Performance Intelligence Quotient

## Competing interests

The authors declare that they have no financial and non-financial competing interests.

## Authors’ contributions

RN drafted the manuscript; EZ helped to draft the manuscript; EM contributed to literature revision and discussion; MEC contributed to write the manuscript; MPR contributed to analyze intellectual and behavioral profile of the patient; GT contributed to analyze intellectual and behavioral profile of the patient; DM contributed to discussion and reviewed the manuscript; CB analyzed intellectual and behavioral profile of the patient; AF contributed to discussion and reviewed the manuscript. All authors read and approved the final manuscript.
